# CMR-based assessment of myocardial edema in the setting of ischemia and reperfusion

**DOI:** 10.1186/1532-429X-15-S1-P212

**Published:** 2013-01-30

**Authors:** Avinash Kali, Andreas Kumar, Richard L Tang, Rohan Dharmakumar

**Affiliations:** 1 Biomedical Imaging Research Institute, Cedars-Sinai Medical Center, Los Angeles, CA, USA; 2 Department of Biomedical Engineering, University of California, Los Angeles, CA, USA; 3 Quebec Heart & Lung Institute, Laval University, Quebec City, QC, Canada

## Background

Cardiovascular Magnetic Resonance (CMR) based assessments of area-at-risk and salvageable myocardium on the basis of myocardial edema in the setting of acute coronary syndrome is of significant clinical interest. However, their dependence on the choice of acquisition method and time to imaging has not been studied. In this study, we investigated the temporal evolution of myocardial edema during ischemia and reperfusion phases using both T2 maps and T2-STIR images.

## Methods

Canines (n=10), subjected to I-R injury, underwent CMR (1.5T) before ischemia (baseline), during ischemia and on days 2, 5, and 7 post-reperfusion. T2-preapred SSFP (T2-preparation durations = 0, 24 and 55 ms; TR/TE = 2.2/1.1ms; BW = 1002 Hz/pixel), T2-STIR (TR = 2-3 RR intervals; TE = 64ms; TI = 170 ms; BW = 355 Hz/pixel) and Late Gadolinium Enhancement (LGE; IR-prepared SSFP; TR/TE = 3.5/1.75 ms; BW = 1002 Hz/pixel) images of the whole LV were acquired. T2 maps were constructed from T2-prepared SSFP images. Using threshold-based analysis, percentage edema volume (*%Edema* from both T2 maps and T2-STIR images), infarct volume (*%Infarct* from LGE images) and salvageable volume (*%Salvage* = *%Edema* - *%Infarct*) were computed relative to total LV myocardial volume.

## Results

**Figure 1:**
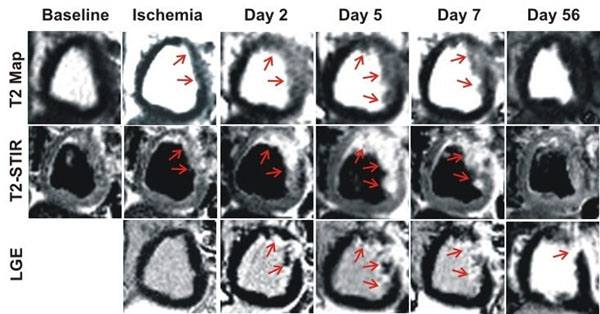
Representative T2 maps, T2-STIR images and LGE images acquired at different study time points from a canine subjected to I-R injury are shown. During ischemia, a small amount of edema was evident on both T2 maps and T2-STIR images (red arrows). Post-reperfusion, extensive edema could be observed at the site of infarction (seen on LGE images; red arrows) on both T2 maps and T2-STIR across days 2, 5 and 7. Edema appeared to have completely regressed by day 56.

**Figure 2:**
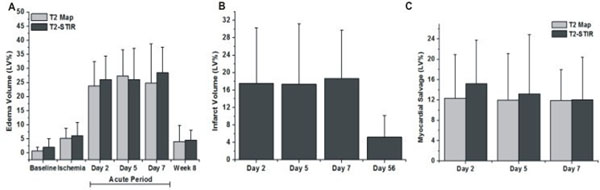
Mean percentage edema (A), infarct (B) and salvageable (C) volumes averaged across all the studies are shown at different time points. During ischemia, there was a small but significant amount of edema relative to baseline (p=0.04). Following reperfusion, edema volume increased nearly 5-fold across days 2, 5 and 7 (p<0.001). Percentage edema, infarct and salvageable volumes remained constant across days 2, 5 and 7 (Edema: p=0.78, Infarct: p=0.21, Salvageable: p=0.72). Percentage edema and infarct volumes returned to baseline levels by day 56 (p<0.001 for both cases). Percentage edema volume during ischemia was significantly lower than both post-reperfusion percentage edema and infarct volumes (p<0.001). There was no difference between percentage edema and salvageable volumes computed by T2 maps and T2-STIR images at any time point.

Representative T2 maps, T2-STIR images and LGE images acquired from a canine at different study points are shown in Figure 1. During ischemia, there was a small but significant increase in *%Edema* relative to baseline (p=0.04; Figure 2A). Post-reperfusion, *%Edema* was elevated nearly 5-fold on days 2, 5 and 7 (p<0.001 for all cases). However, *%Edema* was constant across days 2, 5 and 7 (p=0.78) and returned to baseline levels by day 56 (p=0.02). Both *%Infarct* and *%Salvage* remained unchanged across days 2, 5 and 7 (p = 0.21 and 0.72 respectively; Figures 2B and 2C). Estimates of *%Edema* during ischemia were significantly smaller than the post-reperfusion estimates of *%Edema* or *%Infarct* (p<0.001 for both cases). Estimates of *%Edema* or *%Salvage* obtained at any time point with T2 maps and T2-STIR images were not different (p=0.08 and 0.74 respectively).

## Conclusions

Although ischemia led to a small but significant increase in relative myocardial edema volume, it was not indicative of post-reperfusion infarct or edema volumes. Both relative edema and salvageable myocardial volumes remained unchanged during the sub-acute period of reperfused myocardial infarction. T2 maps and T2-STIR images provided equivalent information regarding relative edema and salvageable volumes.

## Funding

This work was supported in part by grants from American Heart Association (SDG 0735099N) and National Heart, Lung, And Blood Institute (HL091989).

